# Temporal and Cellular Organization of Immune Responses to Live Attenuated Herpes Zoster Vaccination

**DOI:** 10.3390/vaccines14100884

**Published:** 2026-10-08

**Authors:** Junze Wu, Peng-Fei Jin, Liuyu Qin, Yupeng Xue, Mingwei Wei, Guolei Jiang, Zijie Scott Zhang, Rui Cheng, Jing-Xin Li

**Affiliations:** 1 Bio-X Center for Interdisciplinary Innovation, Yunnan University, Kunming 650091, China; 12022130044@mail.ynu.edu.cn (J.W.); 12024114114@stu.ynu.edu.cn (L.Q.); xueyupeng7@gmail.com (Y.X.); zijiezhang@ynu.edu.cn (Z.S.Z.); 2 Yunnan Vaccine Laboratory, Kunming 650000, China; 3 NHC Key Laboratory of Enteric Pathogenic Microbiology, Engineering Research Center of Health Emergency, Jiangsu Province Center for Disease Control and Prevention, Nanjing 210009, China; jpf19891103@163.com (P.-F.J.); js_wmw@163.com (M.W.); 4 School of Public Health, National Vaccine Innovation Platform, Nanjing Medical University, Nanjing 210009, China; 5 School of Science, Institute of Global Health and Emergency Pharmacy, China Pharmaceutical University, Nanjing 211198, China; 6 School of Public Health, Southeast University, Nanjing 210009, China; jgl0223@163.com; 7 Southwest United Graduate School, Kunming 650092, China

**Keywords:** varicella-zoster virus vaccination, single-cell RNA sequencing, longitudinal immune profiling, systems vaccinology, immune aging

## Abstract

**Background:** Cellular responses to live attenuated herpes zoster vaccination and their variation with age remain incompletely characterized. We aimed to define their temporal and cellular organization and identify immune features that differed between age groups. **Methods:** Using a nested cohort design, we evaluated conventional humoral and CD4 T cell responses in 100 adults at Day 0 and Day 30. Olink protein profiles were assessed in 50 participants at Day 0 and Day 1, and longitudinal single-cell RNA sequencing was performed in 18 participants at Day 0, Day 1, and Day 30. **Results:** Vaccination increased gE and VZV antibody levels by Day 30, whereas CD4 T cell cytokine responses were more variable. Olink profiling identified broad early changes shared by both groups and exploratory differences in selected immune and inflammatory proteins. At single-cell resolution, the broadest transcriptional response occurred at Day 1, led mainly by classical monocytes and naive CD4 T cells, followed by a more selective state at Day 30. Differences between age groups involved baseline plasmacytoid dendritic cell abundance and non-classical monocyte regulatory activity, the early conventional dendritic cell type 2 response, and later CD8 T cell and regulatory programs. **Conclusions:** These findings connect conventional immunogenicity with proteomic and cellular responses and identify selected immune states and pathways that differed between age groups across response phases.

## 1. Introduction

Herpes zoster, or shingles, is an infectious disease caused by the reactivation of latent varicella-zoster virus (VZV). Following primary infection during childhood, VZV establishes lifelong latency in sensory ganglia and can reactivate when host immune function declines, leading to the development of herpes zoster [1,2]. Aging is a major risk factor for herpes zoster development and clinical complications, as the incidence of HZ and the risk and severity of postherpetic neuralgia (PHN) increase markedly with advancing age [3,4]. Accumulating evidence indicates that age-associated decline in VZV-specific T cell immunity is a critical immunological mechanism underlying viral reactivation and disease development [5,6,7].

Vaccination has been established as an effective approach for preventing HZ and reducing associated complications. In China, the first domestically approved live attenuated herpes zoster vaccine has been evaluated in a phase III clinical trial. This multicenter, randomized, double-blind, placebo-controlled study demonstrated that the China-developed live attenuated HZ vaccine provided effective protection against HZ in healthy adults aged 40 years and older, with an acceptable safety profile, providing important clinical evidence supporting its application in the Chinese population [8].

Previous studies investigating HZ vaccine-induced immunity have primarily focused on antibody responses and antigen-specific T cell immunity. VZV- and glycoprotein E (gE)-specific antibody titers, as well as VZV-specific T cell responses characterized by the production of activation-associated markers, including IL-2, IFN-γ, TNF-α, and CD40L, have been widely used to evaluate vaccine-induced immune responses [9,10,11,12]. These studies demonstrated that vaccination enhances VZV-specific cellular immunity and promotes the establishment of immune memory responses. However, conventional immunological assays mainly focus on individual immune parameters or overall immune response levels, limiting comprehensive characterization of the dynamic changes in immune cell subsets, cellular interactions, and molecular regulatory networks that drive vaccine-induced immune remodeling. Therefore, these approaches provide limited insights into the underlying mechanisms responsible for vaccine-induced immunity [13].

Recent advances in systems vaccinology have revealed that early transcriptional and metabolic signatures induced after vaccination can reflect innate immune activation states and predict subsequent adaptive immune responses [14,15]. Furthermore, the development of high-dimensional immune profiling technologies has provided new opportunities to dissect vaccine-induced immune responses. Single-cell RNA sequencing (scRNA-seq) has enabled the characterization of dynamic changes in immune cell subsets and cell-type-specific transcriptional programs following vaccination, while high-throughput proteomic platforms allow the detection of early changes in circulating inflammatory mediators and immune regulatory proteins [16,17,18].

We hypothesized that live attenuated herpes zoster vaccination elicits distinct early and later immune responses and that age influences these responses within specific immune cell populations. To investigate this hypothesis, we used three nested analytical cohorts to characterize responses to a live attenuated herpes zoster vaccine developed in China. Conventional gE and VZV antibody measurements and CD4 T cell cytokine measurements in 100 adults were integrated with Olink serum proteomics in 50 participants and longitudinal single-cell RNA sequencing in 18 participants. This design allowed us to characterize conventional immunogenicity, early circulating protein changes, and transcriptional, regulatory, and communication programs within individual cell types, and to examine how these features varied over time and between younger and older participants.

## 2. Materials and Methods

### 2.1. Study Design, Analytical Cohorts and Vaccine Details

The study used three nested analytical cohorts drawn from the same longitudinal vaccination study. The conventional immunogenicity cohort comprised 100 adults, including 70 younger participants aged <70 years and 30 older participants aged ≥70 years, with gE and VZV antibody measurements and VZV responsive CD4 T cell measurements available at Day 0 and Day 30. A subset of 50 participants, including 35 younger and 15 older participants, had paired Olink measurements at Day 0 and Day 1. The longitudinal single-cell cohort comprised 18 participants, including 10 younger and 8 older participants, with an age range of 53–79 years and peripheral blood mononuclear cell samples available at Day 0, Day 1, and Day 30. Participants were recruited in Guanyun County, Jiangsu Province, China, between October and November 2023. Eligible participants were adult men and women aged ≥50 years without a history of herpes zoster infection or prior herpes zoster vaccination. Age was analyzed as a categorical variable, with younger (Y) and older (O) groups defined using a threshold of 70 years. Table S2 summarizes participant characteristics, including age and sex, and sample availability by assay and visit.

The live attenuated herpes zoster vaccine used in this study was Ganwei^®^ (Herpes Zoster Vaccine, Live; NMPA approval number S20233097), manufactured by Changchun BCHT Biotechnology Co., Ltd. (Changchun, China). The vaccine contains live attenuated varicella-zoster virus (VZV) derived from the Oka strain (vOka-BK; GenBank accession no. MF898328.1), propagated in human diploid MRC-5 cells [19]. It is supplied as a lyophilized formulation and reconstituted with the accompanying sterile water for injection immediately before administration. Each 0.5 mL dose contains no less than 4.3 lg PFU of live attenuated VZV (approximately 20,000 PFU). The recommended route is a single 0.5 mL subcutaneous injection into the area below the attachment of the deltoid muscle on the lateral upper arm.

### 2.2. Blood Collection and Sample Processing

Peripheral venous blood was collected at Day 0, Day 1, and Day 30. For participants aged 50–69 years, 23 mL was collected at each of the Day 0 and Day 30 visits, comprising 3 mL in clot activator tubes and 20 mL in EDTA anticoagulant tubes. At Day 1, 13 mL was collected, comprising 3 mL in clot activator tubes and 10 mL in EDTA anticoagulant tubes. For participants aged ≥70 years, 13 mL was collected at each visit, comprising 3 mL in clot activator tubes and 10 mL in EDTA anticoagulant tubes. Blood collected into clot activator tubes was used for serum separation, and anticoagulated blood was used for PBMC isolation. Anticoagulated blood was diluted with RPMI 1640 medium (Gibco, Grand Island, NY, USA) and gently mixed. PBMCs were isolated by density gradient centrifugation using Ficoll lymphocyte separation medium (GE Healthcare, Uppsala, Sweden). Briefly, diluted blood samples were carefully layered onto Ficoll solution at a ratio of 1:1:1 (blood:RPMI 1640 medium:Ficoll) and centrifuged at 1455× *g* for 20 min at room temperature using a swinging-bucket rotor. The PBMC layer at the plasma–Ficoll interface was collected and transferred into new tubes. The isolated PBMCs were washed with RPMI 1640 medium (Gibco, Grand Island, NY, USA) and centrifuged at 524× *g* for 10 min at room temperature. After washing, cells were resuspended in serum-free cryopreservation medium (Yocon, Shanghai, China), subjected to controlled-rate freezing, and stored in liquid nitrogen until further analysis. Written informed consent was obtained from all participants before enrollment.

### 2.3. Antibody Detection

gE-specific IgG antibodies were measured using a commercial ELISA kit (Lvzhubiotech, Beijing, China) with recombinant VZV gE protein as the coating antigen. Serum samples were serially diluted three-fold from an initial dilution of 1:50. Absorbance was measured at 450 nm, and antibody concentrations were determined using a four-parameter logistic (4PL) standard curve and expressed as mIU/mL. Valid dilutions with ≤30% deviation between adjacent dilution points were used for concentration calculation.

VZV-specific IgG antibodies were measured using a commercial ELISA kit (Wuhan Life Technology, Wuhan, China) with inactivated VZV as the coating antigen. Serum or plasma samples were diluted according to the manufacturer’s instructions. Absorbance was measured at 450 nm with a reference wavelength of 620 nm. Antibody concentrations were determined using a 4PL standard curve and expressed as mIU/mL.

### 2.4. gE-Specific T Cell Responses by Spectral Flow Cytometry

Cells were stained with Zombie UV viability dye (BioLegend, San Diego, CA, USA) at room temperature for 40 min, followed by washing with cell staining buffer (PBS containing 0.5% bovine serum albumin (BSA)). Surface staining was performed at 4 °C for 40 min using antibodies against CD3, CD4, and CD8. Cells were then fixed and permeabilized using BD Cytofix/Cytoperm solution (BD Biosciences, San Jose, CA, USA) at 4 °C for 40 min, followed by washing with BD Perm/Wash buffer. Intracellular staining was performed at room temperature for 40 min using antibodies against IFN-γ and IL-2.

Samples were acquired using an Agilent NovaCyte Opteon spectral flow cytometer (Agilent, Santa Clara, CA, USA). Flow cytometry data were analyzed using FlowJo software (version 10.10.0; BD Biosciences). FlowJo was used for manual gating and extraction of flow cytometry parameters. Statistical analyses and visualization were performed using R software (version 4.3.1). The gating strategy is shown in Figure S1, and the antibody panels used for staining are detailed in Table S1.

### 2.5. Conventional Immunogenicity Data Analysis

gE and VZV antibody levels and the frequencies of CD4 T cells producing IFNγ or IL2 after VZV stimulation were available at Day 0 and Day 30 for all 100 participants. For the cellular readouts, the response frequency was calculated as the stimulated value minus the matched unstimulated control value. Changes within each age group were assessed on the original measurement scale using two-sided paired Wilcoxon signed-rank tests, with *p* values adjusted across the eight prespecified comparisons using the Benjamini–Hochberg method. For direct comparisons between age groups, antibody changes were calculated as log2[(Day 30 + 1)/(Day 0 + 1)], whereas CD4 T cell changes were calculated as the absolute Day 30 minus Day 0 difference. Values at Day 0 and Day 30 and the changes calculated for each participant were compared using two-sided Wilcoxon rank-sum tests, with *p* values adjusted across the 12 prespecified comparisons using the Benjamini–Hochberg method.

### 2.6. Single-Cell RNA Sequencing and Data Processing

Cryopreserved PBMC samples were rapidly thawed, washed, and assessed for cell concentration and viability using a Countstar^®^ Rigel fluorescence cell analyzer (Shanghai, China) with acridine orange/propidium iodide (AO/PI) staining. Qualified samples were pooled and adjusted to 2 × 10^5^ cells/mL in PBS for single-cell capture using the Singleron Matrix^®^ (Nanjing, China) single-cell processing system. Single-cell RNA sequencing libraries were generated using the GEXSCOPE^®^ Single Cell RNA Library Kit (Nanjing, China) according to the manufacturer’s instructions and sequenced on an Illumina NovaSeq 6000 (San Diego, CA, USA) platform with 150 bp paired-end sequencing at Singleron Biotechnologies (Nanjing, China).

Raw sequencing data were processed using the CeleScope pipeline (Singleron Biotechnologies). Reads were aligned to the human reference genome using STAR [20] to generate gene expression matrices. For pooled samples, individual sample demultiplexing was performed using souporcell [21], and genotype information derived from whole-genome sequencing (WGS) data was integrated with PLINK-based [22] genetic relatedness analysis to assign cells to individual donors and resolve sample identities.

### 2.7. Single-Cell RNA-Seq Data Processing, Quality Control, and Cell Annotation

Gene expression matrices from individual samples were analyzed in R (version 4.3.1) using Seurat (version 5.3.0) [23]. Only genes shared across all sample matrices were retained before merging. The proportions of mitochondrial, ribosomal protein, and hemoglobin transcripts were calculated for quality control. Cells with >500 detected genes and <20% mitochondrial transcripts were retained; the hemoglobin percentage was inspected but was not used as an exclusion criterion. Doublets were identified independently within each sequencing library using DoubletFinder (version 2.0.4) [24]. The expected doublet rate was 7.5%, pN was set to 0.25, and the first 20 principal components were used. Only cells classified as singlets were retained, after which hemoglobin, mitochondrial, and ribosomal protein genes were removed from the expression matrix.

After quality control, expression values were log-normalized with Seurat NormalizeData, variable genes were selected with the variance-stabilizing transformation method, and the variable gene matrix was scaled before principal component analysis. Technical variation was corrected with Harmony using the composite sequencing-library/participant batch variable. Harmony dimensions 1–20 were used to build the shared nearest neighbor graph and Uniform Manifold Approximation and Projection (UMAP). Broad B cells, T/NK cells, and myeloid compartments were assigned from canonical marker expression. Each compartment was then renormalized, corrected with Harmony, reclustered, and manually annotated using lineage and subtype markers. Clusters with incompatible lineage marker profiles were removed before the final labels were assigned. B lineage and myeloid cells were reclustered at a resolution of 0.5, whereas T/NK cells were reclustered at a resolution of 1. The annotated compartments were subsequently merged and processed using the same normalization, variable feature selection, scaling, principal component analysis, and Harmony workflow. The final neighbor graph and UMAP used Harmony dimensions 1–20 and a clustering resolution of 1.

### 2.8. Cell Abundance Analysis

Cell abundance was calculated for each participant, visit, and annotated cell state with speckle (version 1.2.0) [25]. Proportions were transformed as the arcsine square root using getTransformedProps; cells labeled “unknown” were excluded. Longitudinal changes were tested within each cell state for Day 0 versus Day 1, Day 0 versus Day 30, and Day 1 versus Day 30 using two-sided paired Wilcoxon signed-rank tests. For analyses stratified by age, values at each visit and changes from baseline calculated for each participant were compared between groups using two-sided Wilcoxon rank-sum tests. A comparison was performed when at least three complete participant pairs were available. *p* values were adjusted for multiple testing using the Benjamini–Hochberg (BH) method.

### 2.9. Pseudobulk Differential Gene Expression Analysis

Differential expression was performed separately for each annotated cell state. Raw RNA counts were summed across cells belonging to the same participant, visit, and cell state using a sparse sample design matrix. Samples from a participant at a given visit that contained fewer than five cells were excluded. A cell state entered the analysis only when at least eight participants had eligible samples at all three visits.

The aggregated count matrices were analyzed with edgeR (version 4.0.16) [26]. Lowly expressed genes were removed with filterByExpr, using visit as the grouping variable and min.count = 10, min.total.count = 15, large.n = 10, and min.prop = 0.7. Library sizes were normalized by the trimmed mean of M-values method. Robust dispersion estimates and quasi-likelihood fits were obtained with the design ~ participant + visit, thereby accounting for repeated measurements from the same participant. Quasi-likelihood *F* tests were used for Day 1 versus Day 0, Day 30 versus Day 0, and Day 30 versus Day 1. Genes with a BH-adjusted *p* value < 0.05 and |log2 fold change| ≥ 1 were considered differentially expressed.

### 2.10. Gene Ontology and Blood Transcription Module Analyses

Gene Ontology (GO) Biological Process enrichment was performed for the two cell states with the largest cumulative number of differentially expressed genes across the three visit contrasts. Upregulated and downregulated genes were analyzed separately for each contrast. Groups containing fewer than 10 differentially expressed genes were not tested. For each cell state, the background comprised all genes that passed expression filtering and entered its edgeR models. Enrichment was performed with compareCluster and enrichGO using human gene symbols, BH adjustment, pvalueCutoff = 0.05, and qvalueCutoff = 0.20 from the clusterProfiler package (version 4.10.1) [27].

Single-cell rank-based gene set enrichment analysis (ssGSEA) was performed using the irGSEA package (version 3.3.2) [28]. Blood transcription modules (BTMs) were used as reference gene sets [29]. Entries labeled “TBA” were removed, member genes were converted to unique symbols, and modules represented by fewer than five genes in the single-cell expression matrix were excluded. Enrichment scores were calculated for each cell using a Kolmogorov–Smirnov-based ssGSEA approach and were averaged within each participant, cell state, and visit. Longitudinal differences were assessed using two-sided paired Wilcoxon signed-rank tests, and age group differences were assessed using two-sided Wilcoxon rank-sum tests. *p* values were adjusted for multiple testing using the BH method.

### 2.11. Temporal Gene Expression Clustering

Temporal coexpression patterns were derived separately for each cell state from log-normalized RNA expression with the ClusterGVis package (version 0.1.4) [30]. Profiles containing fewer than 20 cells for a participant at a given visit were excluded, and at least three eligible profiles were required at each visit. Genes had to be detected in at least 10% of the retained cells and show nonzero variation across Day 0, Day 1, and Day 30. For each gene, expression variance across the three visit means was calculated, and the 2000 genes with the highest variance were retained. Expression was first averaged within each participant and visit and then across participants at each visit, generating a matrix with genes as rows and visits as columns.

The matrix was standardized by gene and grouped into four temporal expression patterns with Mfuzz through clusterData. GO Biological Process enrichment was performed for each cluster using enrichCluster with pvalueCutoff = 0.05.

### 2.12. T Cell Functional State Analysis

Ten T cell functional state signatures supplied by TCellSI (version 1.2.0) [31] were scored in nine annotated T and NKT cell states from log-normalized RNA expression using Seurat AddModuleScore. Scores were averaged for each participant within each cell state and visit. Analyses were restricted to Day 0 and Day 30. Longitudinal differences within each age group were assessed using two-sided paired Wilcoxon signed-rank tests, and differences between age groups at each visit were assessed using two-sided Wilcoxon rank-sum tests. *p* values were adjusted using the Benjamini–Hochberg method. For visualization, DeltaScore was calculated as the mean score in the first condition minus that in the second condition shown for each comparison.

### 2.13. Hallmark Gene Set Enrichment Analysis

Within each T cell state, gene expression was compared between cells from older and younger participants at Day 30 using the Wilcoxon rank-sum test implemented in Seurat FindMarkers. All tested genes were retained and ranked by average log2 fold change. Preranked gene set enrichment analysis was performed using clusterProfiler with human Hallmark gene sets obtained through msigdbr (version 26.1.0) [32]. Enrichment was summarized using the normalized enrichment score and BH-adjusted *p* value. A positive normalized enrichment score indicated enrichment in the older group, whereas a negative score indicated enrichment in the younger group.

### 2.14. B Cell Functional Module Scoring

Eight B cell functional gene signatures were selected from the collection reported by Yang et al. [33]. These signatures corresponded to MSigDB gene sets representing MYC targets V2, the G1/S and G2/M transitions, cellular stress, responses to interferon gamma and type I interferon, antigen processing and presentation, and B cell activation. Signature scores were calculated in naive B cells, memory B cells, and plasma cells using Seurat AddModuleScore. Scores were averaged for each participant, cell state, and visit. Day 30 and Day 0 were compared within each age group using two-sided paired Wilcoxon signed-rank tests, whereas the two age groups were compared at Day 0 and Day 30 using two-sided Wilcoxon rank-sum tests. *p* values were adjusted for multiple testing using the Benjamini–Hochberg method. DeltaScore represented the mean score difference in the direction shown for each comparison.

### 2.15. Cell–Cell Communication Analysis

Cell–cell communication was inferred from normalized RNA expression using CellChat (version 2.1.2) [34] and the human CellChat database, with annotated cell states used to define the interacting populations. Separate networks were constructed for the younger and older groups at Day 0, Day 1, and Day 30. Overexpressed signaling genes and ligand–receptor interactions were identified, followed by calculation of abundance-adjusted communication probabilities, pathway-level probabilities, and aggregate interaction networks. The six networks were then merged for comparison. Early analyses compared Day 0 and Day 1 information flow and MHC-II signaling from cDC2. Later analyses compared Day 0 and Day 30 ligand–receptor communication directed to regulatory CD4 T cells, communication originating from naive B cells, and the CD86 signaling network.

### 2.16. Regulon Analysis

Regulon activity was calculated using pySCENIC (version 0.12.1) [35]. Regulatory network inference was performed on Day 0 and Day 1 cells from the innate immune populations and on Day 0 and Day 30 cells from the T cell populations using raw UMI counts. Genes were retained when detected in at least the larger of 20 cells or 1% of input cells and when their total count was at least three times that minimum cell number. A loom file containing the retained cells and genes was analyzed using the three-step GRNBoost2, cisTarget, and AUCell workflow. Motif pruning used the hg38 RefSeq-r80 v10 clustered ranking databases covering ±10 kb around the transcription start site and the −500/+100 bp promoter interval, together with the matching v10 non-redundant HGNC motif annotations. Regulons were required to contain at least 20 genes during the cisTarget step, and AUCell scores were calculated for the input cells.

Regulon AUC values were averaged within each participant, visit, and cell state, and regulons with at least 10 target genes were retained. Innate immune populations were compared between age groups at Day 0, Day 1, and for the Day 1 minus Day 0 change calculated for each participant. T cell populations were compared at Day 0, Day 30, and for the Day 30 minus Day 0 change. Two-sided Wilcoxon rank-sum tests were used, and *p* values were adjusted for multiple testing using the BH method.

### 2.17. Serum Proteomic Profiling Using Olink Target 96 Panels

Serum protein levels were measured using the Olink Target 96 Inflammation Panel and Immune Response Panel (Olink Biosciences, Uppsala, Sweden), each comprising 92 proteins, based on the proximity extension assay (PEA) technology. Serum samples were analyzed according to the manufacturer’s instructions, and protein abundance was quantified using the Signature Q100 microfluidic qPCR platform (Olink Biosciences, Uppsala, Sweden). Raw data were processed using Olink NPX Signature (version 2.0) software for quality control and normalization, including internal control normalization and inter-plate calibration. Protein expression levels were reported as normalized protein expression (NPX) values on a log2 scale. Proteins with poor detection quality were excluded based on the limit of detection (LOD) and missing frequency. Quality control analyses, including principal component analysis (PCA), sample correlation analysis, and hierarchical clustering, were performed on the normalized NPX dataset before downstream analyses.

### 2.18. Olink Protein Analysis

Paired normalized protein expression (NPX) data at Day 0 and Day 1 were available for the Olink cohort of 50 participants from the Olink Target 96 Immune Response and Target 96 Inflammation panels. NPX was analyzed on its native log2 scale. Sample level quality control flags were audited before analysis, and no sample classified as failed was included. Within each panel, proteins were retained when at least 50% of measurements had NPX at or above the limit of detection for the corresponding plate.

Each panel was analyzed separately with OlinkAnalyze (version 4.5.0) [36]. For every retained protein, a linear mixed model equivalent to NPX ~ age group ∗ visit + (1|participant) was fitted with olink_lmer. Post hoc contrasts from the model estimated differences between age groups at Day 0 and Day 1 and the Day 1 minus Day 0 response within each age group. The difference in response between age groups was defined as (O_Day1 − O_Day0) − (Y_Day1 − Y_Day0) and was evaluated using the interaction between age group and visit. *p* values were adjusted for multiple testing using the BH method within each panel and contrast. The NPX values, the LOD audit, and complete model statistics are provided in Table S10.

### 2.19. Statistical Analyses

All statistical analyses were performed in R (version 4.3.1). Longitudinal comparisons within participants were evaluated using two-sided paired Wilcoxon signed-rank tests, whereas comparisons between age groups were evaluated using two-sided Wilcoxon rank-sum tests. Cliff’s delta was calculated for age group comparisons of BTM scores and regulon activity as a rank-based effect size. Positive values indicate higher scores in the older group, and negative values indicate higher scores in the younger group. Pseudobulk differential expression was assessed using edgeR quasi-likelihood *F* tests, and Olink data were analyzed using linear mixed models with participant as a random intercept. *p* values were adjusted for multiple testing using the BH method when applicable, and adjusted *p* values < 0.05 were considered statistically significant. Participants were treated as independent biological replicates.

## 3. Results

### 3.1. Study Cohorts and Conventional Immune Responses

**Figure 1:**
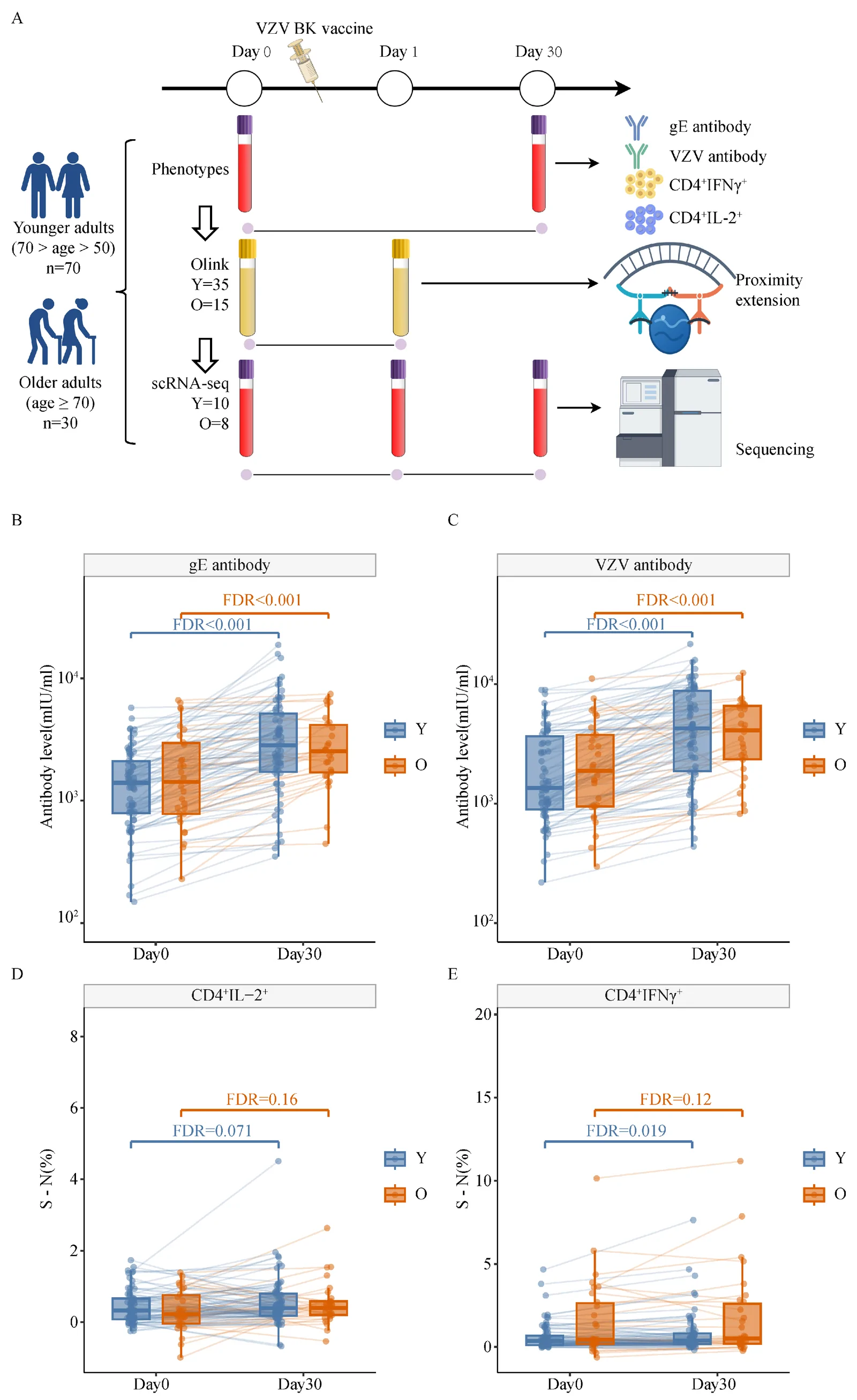
Nested cohort design and conventional immune responses following VZV vaccination. (**A**) Study design. Conventional immune measurements were available at Day 0 and Day 30 for 100 participants (Y, *n* = 70; O, *n* = 30), Olink measurements were available at Day 0 and Day 1 for 50 participants (Y, *n* = 35; O, *n* = 15), and single-cell RNA sequencing was performed at Day 0, Day 1, and Day 30 for 18 participants (Y, *n* = 10; O, *n* = 8). (**B**) gE antibody levels. (**C**) VZV antibody levels. (**D**) Frequencies of gE-specific IL2^+^ CD4T cells. (**E**) Frequencies of gE-specific IFNγ^+^ CD4 T cells. Cytokine frequencies are shown after subtraction of the corresponding unstimulated control values. In (**B**–**E**), lines connect paired observations, and boxes show the median and interquartile range. Comparisons between Day 0 and Day 30 within each age group were performed using two-sided paired Wilcoxon signed-rank tests with Benjamini–Hochberg correction. FDR, false discovery rate; gE, glycoprotein E; VZV, varicella-zoster virus; IL2, interleukin 2; IFNγ, interferon gamma; Y, younger participants aged <70 years; O, older participants aged ≥70 years. Participant characteristics, individual immune measurements, and paired comparison statistics are provided in Table S2.

Three nested cohorts were used to examine the response at increasing molecular resolution (Figure 1A). The conventional immunogenicity cohort included 100 participants, with 70 in the younger group and 30 in the older group. The Olink subset included 50 participants, with 35 in the younger group and 15 in the older group. The single-cell RNA sequencing subset included 18 participants, with 10 in the younger group and 8 in the older group.

In the cohort of 100 participants, gE antibody levels increased from Day 0 to Day 30 in both younger and older participants (FDR = 4.63 × 10^−12^ and 3.34 × 10^−4^, respectively; Figure 1B). VZV antibody levels also increased in both groups (younger, FDR = 2.91 × 10^−12^; older, FDR = 1.04 × 10^−4^; Figure 1C). Following gE stimulation, the frequency of CD4 T cells producing IL2 did not increase significantly from Day 0 to Day 30 in either group (younger, FDR = 0.0713; older, FDR = 0.162; Figure 1D). The frequency of CD4 T cells producing IFNγ increased significantly in younger participants (FDR = 0.0189), whereas the increase in older participants was not statistically significant (FDR = 0.119; Figure 1E; Table S2).

### 3.2. Single-Cell Profiling Resolves Peripheral Immune Cell Changes Following VZV Vaccination

**Figure 2:**
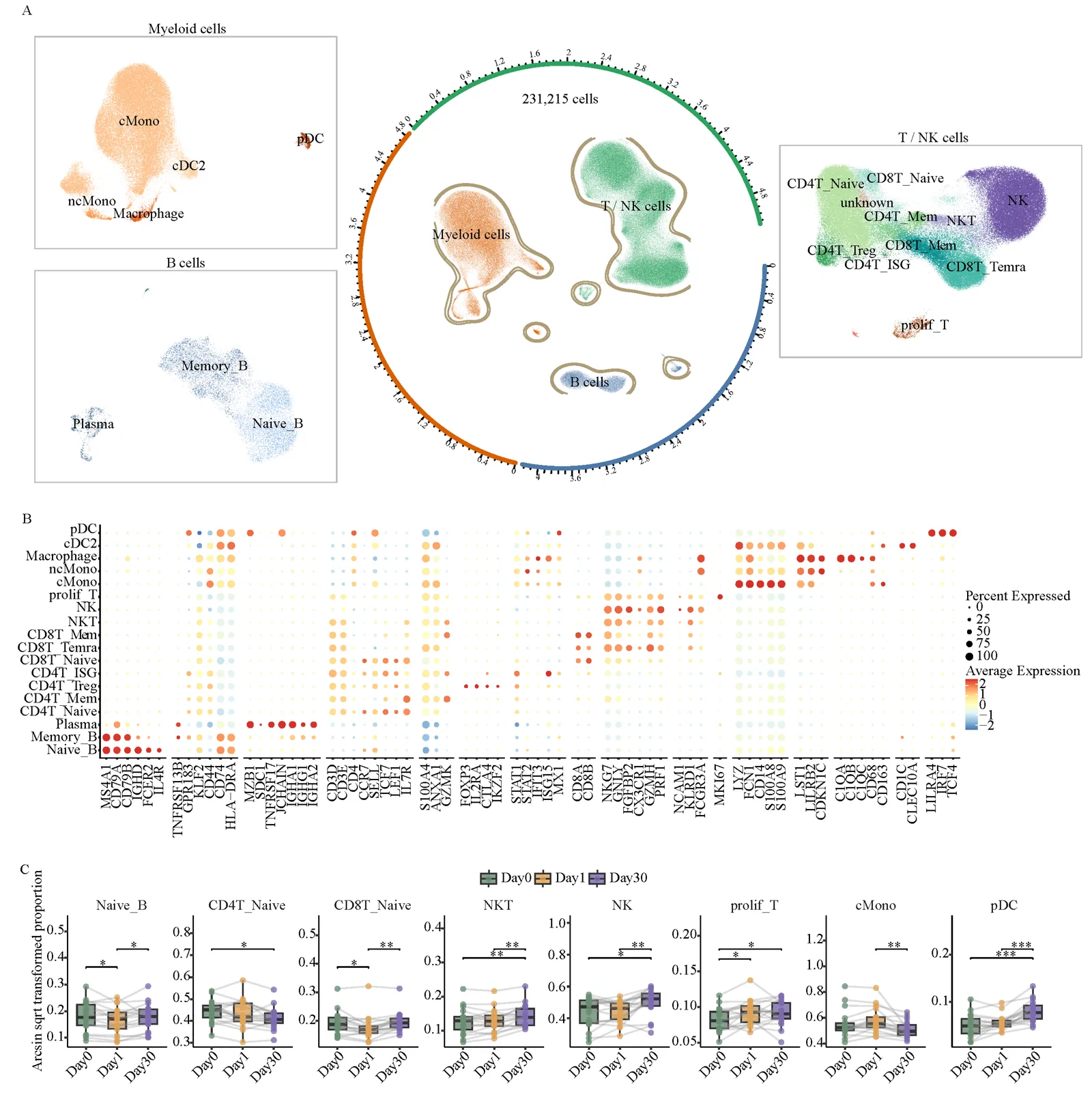
Single-cell profiling and temporal changes in circulating immune cell abundance following VZV vaccination. (**A**) Integrated UMAP of 231,215 peripheral blood mononuclear cells and separate UMAPs for the B, T/NK, and myeloid lineages, showing 18 annotated immune cell states. (**B**) Expression of canonical lineage and subtype marker genes. Dot size represents the percentage of cells expressing each gene, and color represents scaled average expression. (**C**) Abundance of eight cell states with at least one significant visit comparison in 18 participants at Day 0, Day 1, and Day 30. Proportions were arcsine square root transformed. Points represent participants, gray lines connect paired observations, and boxes show the median and interquartile range. Differences between visits were assessed using two-sided paired Wilcoxon signed-rank tests with Benjamini–Hochberg correction. * FDR < 0.05, ** FDR < 0.01, and *** FDR < 0.001. UMAP, uniform manifold approximation and projection; FDR, false discovery rate; cMono, classical monocytes; ncMono, non-classical monocytes; cDC2, conventional dendritic cell type 2; pDC, plasmacytoid dendritic cells; NK, natural killer cells; NKT, natural killer T cells; Naive, naive T cells; Mem, memory T cells; Temra, terminally differentiated effector memory T cells; Treg, regulatory T cells; ISG, interferon-stimulated gene; prolif_T, proliferating T cells.

We then characterized the vaccination time course at single-cell resolution in 18 adults, including 10 participants in the younger group and 8 in the older group (Table S2). Peripheral blood mononuclear cells were analyzed at Day 0, Day 1, and Day 30. After quality control, the dataset comprised 231,215 cells. T cells and natural killer cells formed the largest lineage (144,711 cells), followed by myeloid cells (70,201 cells) and B lineage cells (15,216 cells). Unsupervised embedding and annotation within each lineage resolved 18 immune states, of which classical monocytes (62,900 cells), natural killer cells (47,504 cells), and naive CD4 T cells (40,221 cells) were the most abundant (Figure 2A). The expression patterns of canonical lineage and subtype marker genes are shown in Figure 2B.

Paired comparisons showed several patterns in cell abundance across the three visits. Naive B cells decreased from Day 0 to Day 1 and recovered between Day 1 and Day 30. Naive CD8 T cells followed a similar early decrease and subsequent recovery. By Day 30, naive CD4 T cell abundance was lower than at baseline, whereas natural killer T (NKT) and NK cells were more abundant than at both Day 0 and Day 1. Proliferating T cells increased at Day 1 and remained above baseline at Day 30. Among myeloid and dendritic cell populations, classical monocytes decreased between Day 1 and Day 30, while plasmacytoid dendritic cell (pDC) abundance was higher at Day 30 than at either Day 0 or Day 1 (Figure 2C). The complete abundance profiles and paired statistics are shown in Figure S2, and participant-level transformed abundances are provided in Table S3. These trajectories resolved three abundance motifs: early contraction followed by recovery, persistent elevation after vaccination, and delayed Day 30 expansion.

### 3.3. Vaccination Drives Distinct Early and Late Immune Responses

**Figure 3:**
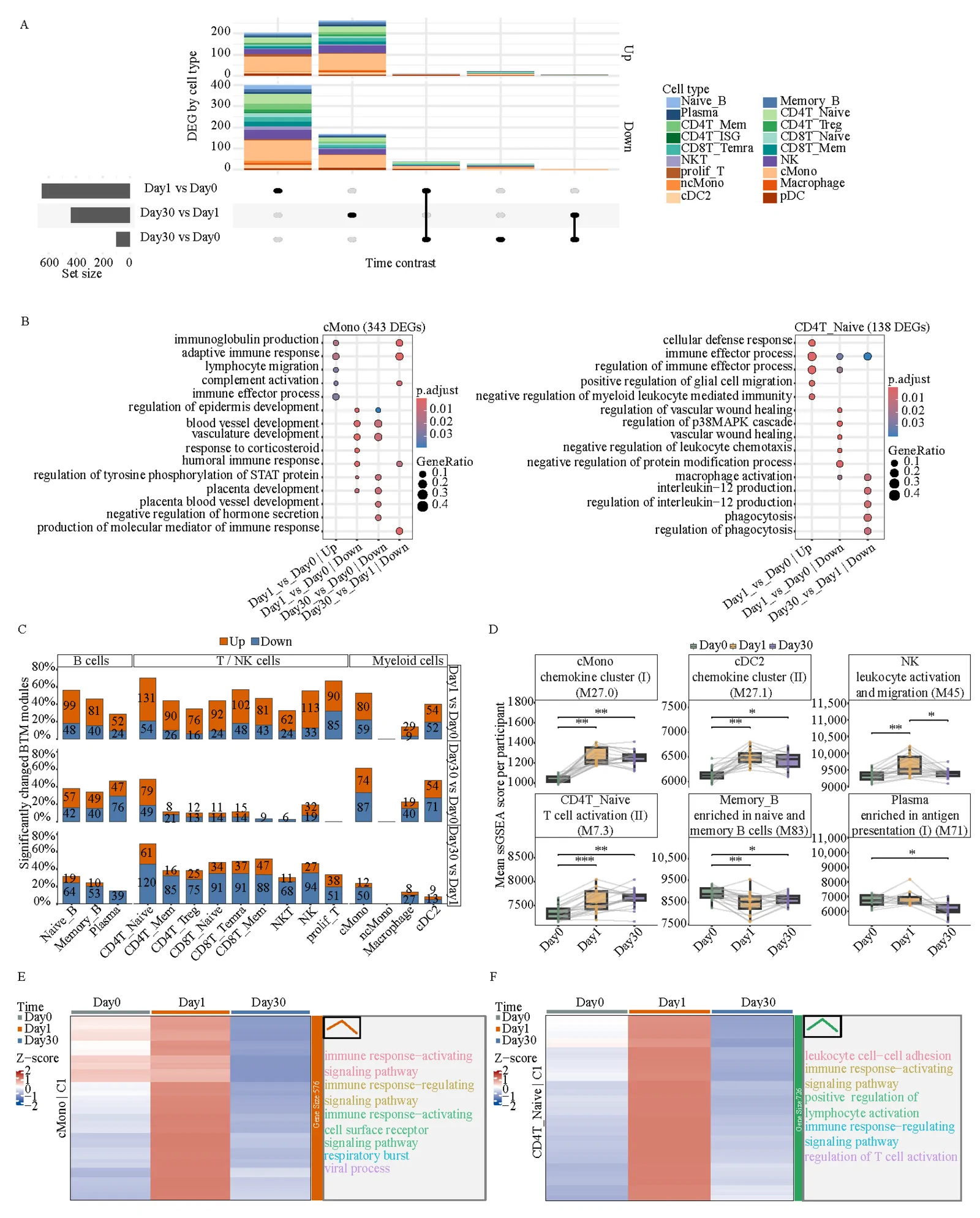
Temporal transcriptional responses to VZV vaccination across immune cell states. (**A**) UpSet plots showing the overlap of differentially expressed gene (DEG) events among Day 1 versus Day 0, Day 30 versus Day 0, and Day 30 versus Day 1. Upregulated and downregulated events are displayed separately. A gene was defined as a DEG if it met the thresholds of FDR < 0.05 and |log2 fold change| ≥ 1; bars are colored by cell state, and connected dots indicate the visit comparisons included in each intersection. (**B**) Gene Ontology Biological Process enrichment of DEGs in classical monocytes and naive CD4 T cells, the two cell states with the largest cumulative DEG counts. Up to five terms are shown for each available combination of visit comparison and direction. Dot size represents the GeneRatio, and color represents the adjusted *p* value. (**C**) Fractions of the 263 blood transcription modules (BTMs) with significantly higher or lower scores in each cell state for the three visit comparisons. Numbers within the bars indicate significant module counts. BTM scores were considered significant at FDR < 0.05. (**D**) Participant-level mean ssGSEA scores for six representative cell states. Points represent participants, gray lines connect paired observations, and boxes show the median and interquartile range. Differences between visits were assessed using paired Wilcoxon signed-rank tests with BH correction. * FDR < 0.05, ** FDR < 0.01, and *** FDR < 0.001. (**E**) Temporal expression pattern of a 576 gene cluster in classical monocytes. (**F**) Temporal expression pattern of a 726 gene cluster in naive CD4 T cells. Heatmaps show standardized expression across visits, with average expression profiles and GO Biological Process annotations.

We next examined the overall transcriptional response to vaccination using paired pseudobulk differential expression analysis for each cell type. At FDR < 0.05 and |log2FC| ≥ 1, the Day 1 versus Day 0 comparison yielded 649 differentially expressed gene (DEG) events across individual cell types, including 213 increases and 436 decreases. The Day 30 versus Day 0 comparison yielded 100 events (33 increased and 67 decreased), whereas 434 events were detected between Day 30 and Day 1 (263 increased and 171 decreased; Figure 3A). Classical monocytes contributed the largest cumulative number of events across the three contrasts (343), followed by naive CD4 T cells (138) and NK cells (133; Table S4). Only 100 DEG events were detected at Day 30 versus Day 0, compared with 649 at Day 1 versus Day 0. This reduction indicates that the broad early transcriptional response had largely subsided by Day 30, although a smaller set of transcriptional differences remained relative to baseline.

Because classical monocytes and naive CD4 T cells had the largest numbers of differentially expressed genes, we further performed Gene Ontology Biological Process enrichment analysis for these two cell types. In classical monocytes, genes upregulated at Day 1 were enriched for immunoglobulin production, adaptive immune responses, lymphocyte migration, complement activation, and immune effector processes. Genes downregulated in the same comparison were enriched for vascular development, humoral immune responses, and regulation of epidermis development. In naive CD4 T cells, genes upregulated at Day 1 were enriched for immune effector and cellular defense processes. Other contrasts included terms related to vascular wound healing, negative regulation of leukocyte chemotaxis, interleukin-12 production, and phagocytosis (Figure 3B; Table S5). Together, these patterns suggest that vaccination elicited an early response that linked innate immune functions in monocytes with lymphocyte recruitment and adaptive immune coordination, whereas later changes involved immune regulation, chemotaxis, and wound healing.

Blood transcription module analysis further revealed coordinated temporal changes across multiple gene modules involved in immune responses after vaccination. Across the 263 modules screened in each cell type, 1817 combinations of cell types and modules differed between Day 1 and Day 0 (1232 higher and 585 lower). The corresponding totals were 958 for Day 30 versus Day 0 (457 higher and 501 lower) and 1363 for Day 30 versus Day 1 (354 higher and 1009 lower; Figure 3C; Table S6). Representative modules resolved three broad trajectories (Figure 3D). Sustained increases were observed in chemokine module M27.0 in classical monocytes, chemokine module M27.1 in cDC2, and activation module M7.3 in naive CD4 T cells; each remained higher than baseline at both visits after vaccination. Leukocyte activation and migration module M45 in NK cells showed a transient increase at Day 1, followed by a decline at Day 30 and no detectable difference between Day 30 and baseline. Conversely, module M83 in memory B cells was lower at both visits after vaccination, and antigen presentation module M71 in plasma cells was lower at Day 30. Together, these trajectories revealed a temporally structured response comprising sustained myeloid chemokine programs, activation programs in naive CD4 T cells, a transient Day 1 response in NK cells, and lower scores for selected modules in memory B cells and plasma cells.

Temporal coexpression patterns further localized the dominant early response. Among the four clusters identified from the 2000 genes showing the greatest variation across visits in classical monocytes, one contained 576 genes and showed highest standardized expression at Day 1, followed by a marked decline at Day 30. This cluster was annotated with immune response signaling, respiratory burst, and viral process terms (Figure 3E). A separate cluster of 726 genes in naive CD4 T cells followed the same pattern centered on Day 1 and was enriched for leukocyte adhesion, immune response signaling, lymphocyte activation, and regulation of T cell activation (Figure 3F). Together, these patterns identified Day 1 as the principal phase of the transcriptional response, characterized by concurrent induction of effector programs in classical monocytes and activation programs in naive CD4 T cells, both of which had markedly declined by Day 30.

### 3.4. Age Differences in Early Innate Immune Responses to Vaccination

**Figure 4:**
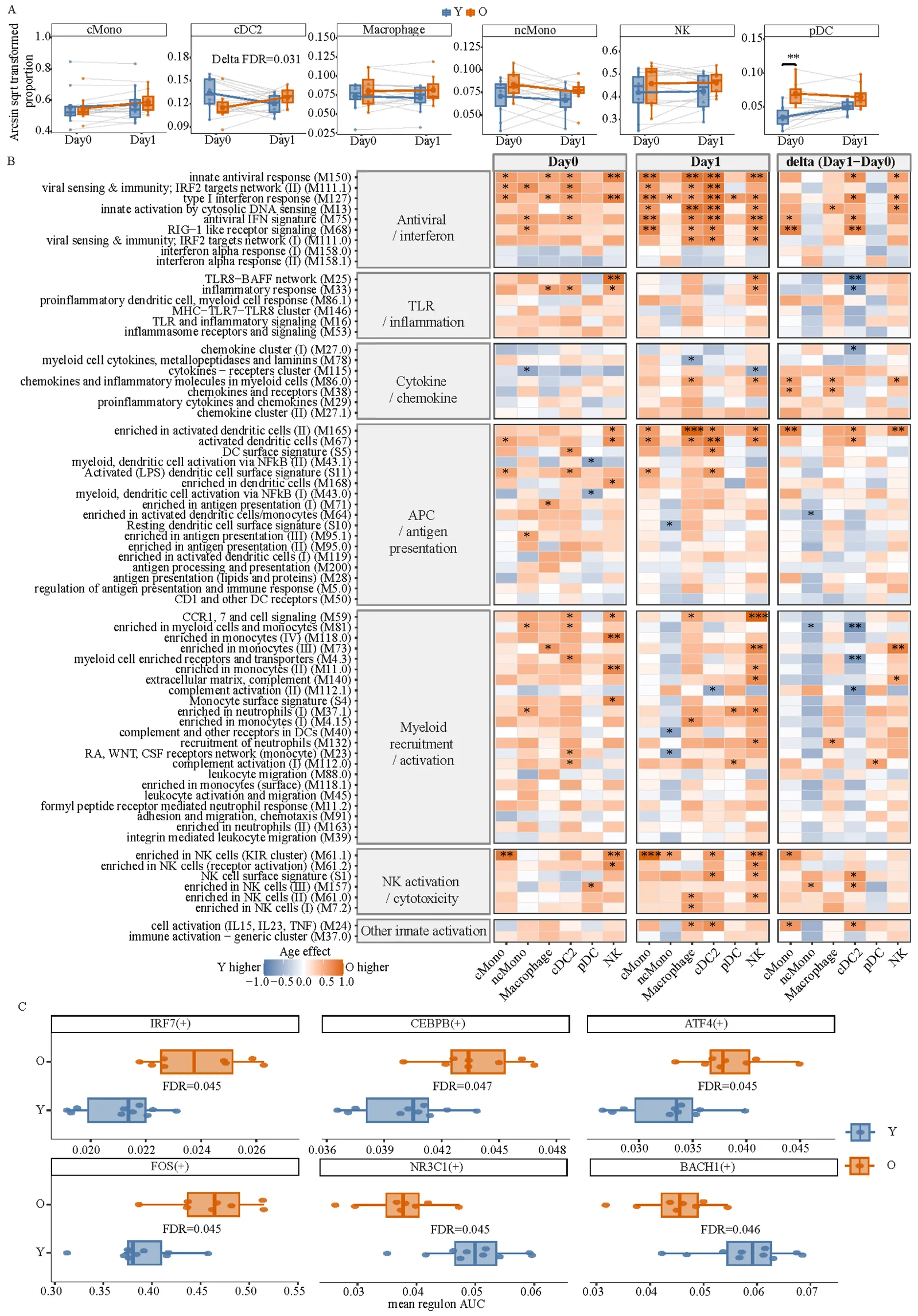
Innate immune features across age groups after VZV vaccination. (**A**) Abundance of six innate immune cell states at Day 0 and Day 1 in younger (Y; *n* = 10) and older (O; *n* = 8) participants. Proportions were arcsine square root transformed. Differences between age groups at each visit and in Day 1 minus Day 0 changes were assessed using Wilcoxon rank-sum tests with BH correction. * FDR < 0.05, ** FDR < 0.01, and *** FDR < 0.001. (**B**) Cliff’s delta for BTM scores at Day 0, Day 1, and for Day 1 minus Day 0 changes. Positive and negative values indicate higher scores in the older and younger groups, respectively. Stars indicate *p* values: * *p* < 0.05, ** *p* < 0.01, and *** *p* < 0.001. (**C**) Participant mean AUC values for six representative regulons in non-classical monocytes at Day 0. Differences between age groups were assessed using two-sided Wilcoxon rank-sum tests with Benjamini–Hochberg correction. Y, younger participants aged <70 years; O, older participants aged ≥70 years; BTM, blood transcription module; AUC, area under the curve; FDR, false discovery rate; cMono, classical monocytes; ncMono, non-classical monocytes; cDC2, conventional dendritic cell type 2; pDC, plasmacytoid dendritic cells; NK, natural killer cells.

We next assessed whether the two age groups differed in baseline innate immune composition or in the early response to vaccination. At baseline, pDC abundance was higher in the older group than in the younger group (Figure 4A). Because baseline differences do not necessarily indicate differences in vaccine responsiveness, we further compared the changes from Day 0 to Day 1 calculated for each participant. Among the six innate immune cell types examined, only the change in cDC2 abundance differed between the age groups. cDC2 abundance tended to decrease in the younger group but increase in the older group. None of the other innate immune cell types showed a significant difference between age groups in their early abundance changes. Participant-level transformed abundances are provided in Table S7.

Analysis of innate BTM scores revealed transcriptional patterns related to age at baseline, Day 1, and during the early response to vaccination. At Day 0, 46 combinations of modules and cell types had *p* < 0.05. The older group showed higher scores for innate antiviral response module M150 and type I interferon response module M127 in NK cells. Viral sensing and IRF2 target module M111.1 was also higher in cDC2 cells and non-classical monocytes from the older group, indicating that differences between age groups in antiviral and interferon responses were already present before vaccination (Figure 4B).

At Day 1, 71 combinations of modules and cell types had *p* < 0.05. The older group showed a higher score for activated dendritic cell module M165 in macrophages. Higher scores were also observed for innate antiviral response module M150 in macrophages and cDC2 cells and type I interferon response module M127 in cDC2 cells. Thus, the patterns observed at Day 1 involved multiple myeloid programs related to dendritic cell activation, viral sensing, and interferon responses.

For the paired Day 1 minus Day 0 response, 37 combinations of modules and cell types had *p* < 0.05. RIG-I-like receptor signaling module M68 and type I interferon response module M127 increased more prominently in cDC2 cells from the older group, whereas both tended to decrease in the younger group. Conversely, TLR8–BAFF network module M25 and inflammatory response module M33 increased in cDC2 cells from the younger group but decreased in those from the older group (Table S8). These findings suggest that age was associated not only with the antiviral immune state before vaccination but also with the coordination of viral sensing, interferon, and inflammatory signals in cDC2 cells during the early response.

Regulon analysis further identified differences between age groups in the baseline regulatory state of non-classical monocytes. At Day 0, 29 of the 121 regulons analyzed in non-classical monocytes differed significantly between the two age groups after BH correction. Six regulons were selected from this set to represent distinct aspects of immune regulation. FOS, IRF7, ATF4, and CEBPB regulon activities were higher in older participants and were associated with immediate early responses mediated by AP-1, antiviral regulation dependent on type I interferon, the integrated stress response, and myeloid cell functional programming, respectively. In contrast, NR3C1 and BACH1 regulon activities were lower in older participants; these regulons are related to immune homeostasis mediated by glucocorticoids and the regulation of heme and oxidative stress, respectively (Figure 4C). Together, these six regulons indicate that non-classical monocytes from older participants had distinct baseline regulatory features involving immediate early activation, antiviral defense, cellular stress responses, and immune homeostasis. The broader regulon screen is shown in Figure S3, and statistics for all tested regulons are provided in Table S9. No regulon met the FDR threshold at Day 1 or for the Day 1 minus Day 0 response. Collectively, the analyses revealed differences between age groups in baseline pDC abundance and regulatory states in non-classical monocytes, together with differences in the early cDC2 abundance response after vaccination.

To investigate how innate immune features related to age were linked to intercellular signaling, we used CellChat to infer ligand–receptor communication networks in the younger and older groups at Day 0 and Day 1. Comparison of information flow at the pathway level showed that MHC-I, MHC-II, MIF, CD99, GALECTIN, and CLEC constituted prominent communication programs across the four conditions (Figure S4A). MIF information flow decreased from Day 0 to Day 1 in both age groups. In contrast, MHC-II signaling showed different early trajectories, increasing after vaccination in the younger group but decreasing in the older group.

Because cDC2 abundance and transcriptional responses also varied by age group, we further examined the cellular architecture of MHC-II signaling. cDC2 was the leading inferred sender of MHC-II signals in all four networks. At baseline, MHC-II communication originating from cDC2 was broadly similar between the age groups and was directed mainly toward pDCs, macrophages, classical and non-classical monocytes, interferon-responsive CD4 T cells, and cDC2 itself. At Day 1, the inferred network originating from cDC2 became stronger and expanded to include proliferating T cells and plasma cells in the younger group. In the older group, the corresponding network was weaker and included an additional connection to naive CD4 T cells, but not to proliferating T cells or plasma cells (Figure S4B). These patterns suggest that the early cDC2 response may differ with age not only in abundance and transcriptional state but also in its inferred coordination of adaptive immune populations through MHC-II signaling.

### 3.5. Early Circulating Protein Responses in the Olink Cohort

Early circulating protein responses were examined in 50 participants, including 35 in the younger group and 15 in the older group, using the Olink Target 96 Immune Response and Inflammation panels. After filtering by the limit of detection, 84 and 80 proteins were retained, respectively. Both age groups showed broad changes between Day 0 and Day 1. After BH correction, 22 Immune Response and 29 Inflammation proteins changed in the younger group, compared with 24 and 15 proteins in the older group. Fifteen Immune Response proteins and 10 Inflammation proteins changed in both groups, demonstrating a substantial shared early circulating protein response (Table S10).

**Figure 5:**
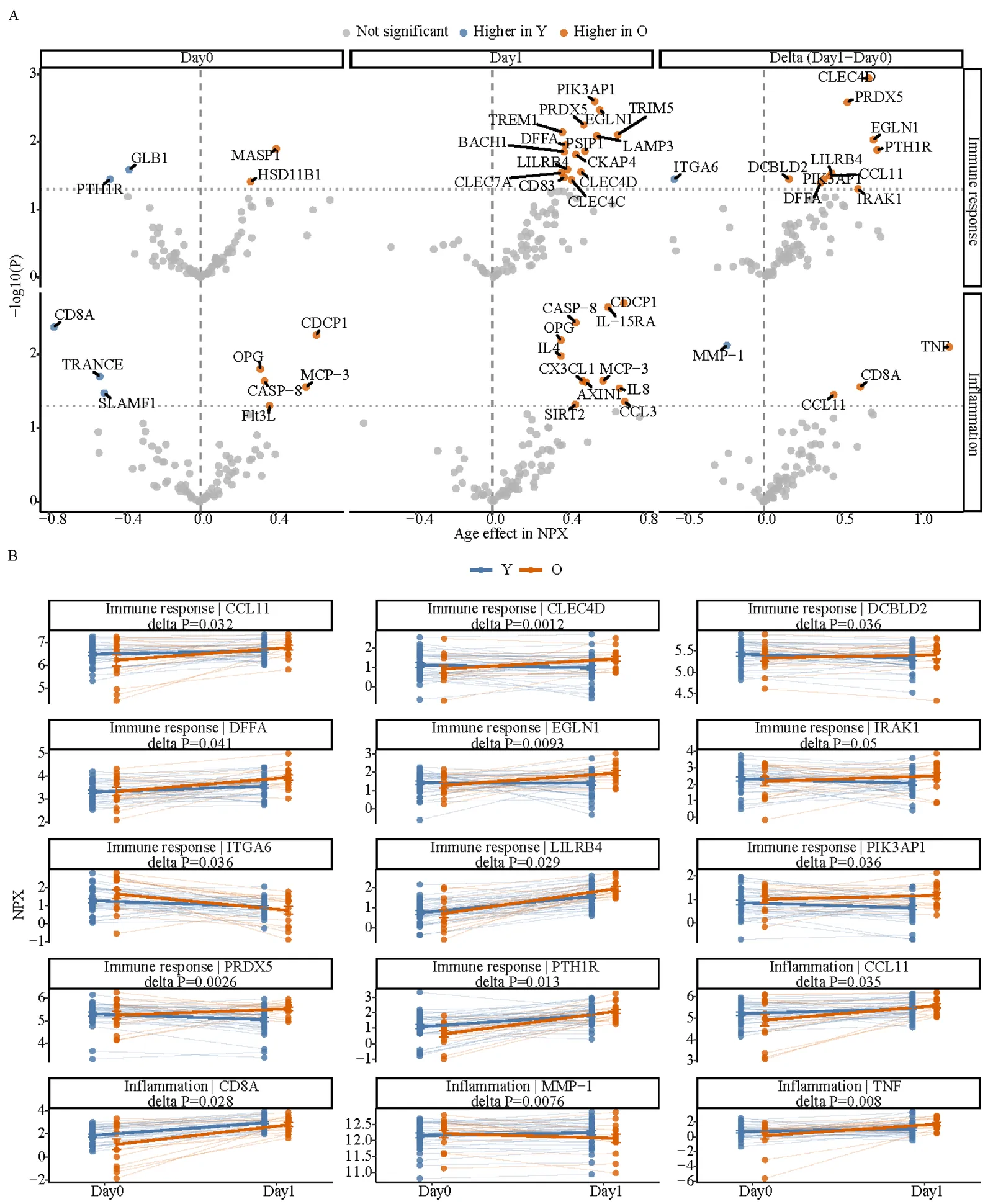
Early circulating protein responses in the Olink cohort following VZV vaccination. (**A**) Volcano plots of normalized protein expression differences between age groups at Day 0 and Day 1 and of the difference in Day 1 minus Day 0 responses. The age effect is O minus Y at each visit and (O_Day1 − O_Day0) − (Y_Day1 − Y_Day0) for the response. Proteins with nominal *p* < 0.05 are colored and labeled. (**B**) Participant trajectories for 15 proteins with a nominal age group by visit interaction *p* < 0.05 in 35 younger and 15 older participants. Thin lines connect paired samples, and thick lines with error bars show the group mean and SEM. *p* values were obtained from linear mixed models. None of the displayed interactions remained significant after BH correction.

Exploratory comparisons also identified protein patterns that differed between age groups. At Day 0, 4 Immune Response and 8 Inflammation proteins had nominal *p* < 0.05. At Day 1, the corresponding counts increased to 15 and 11, and all 26 proteins had higher NPX values in older participants (Figure 5A). These proteins included components related to innate recognition and immune signaling, such as TREM1, CLEC4D, CLEC7A, PIK3AP1, and LILRB4, as well as proteins linked to antiviral defense, redox regulation, and inflammation, including TRIM5, PRDX5, EGLN1, CASP-8, IL8, and CCL3. None of these age group contrasts remained significant after BH correction.

The age group by visit interaction had nominal *p* < 0.05 for 15 proteins, including 11 from the Immune Response panel and 4 from the Inflammation panel (Figure 5B). Thirteen interaction estimates were positive, indicating that the Day 1 minus Day 0 responses were higher in older participants than in younger participants, whereas ITGA6 and MMP-1 showed the opposite direction. The candidates included CLEC4D, LILRB4, PIK3AP1, and IRAK1, which participate in innate immune recognition or signaling; PRDX5 and EGLN1, which are related to redox and oxygen sensing; and TNF, CCL11, and CD8A, which reflect inflammatory or lymphocyte-associated processes. No interaction remained significant after BH correction. The Olink results therefore identified a broad shared early protein response and an exploratory set of age group differences involving innate, inflammatory, and cellular stress pathways.

### 3.6. Age Is Associated with Selected Late Adaptive Immune States

**Figure 6:**
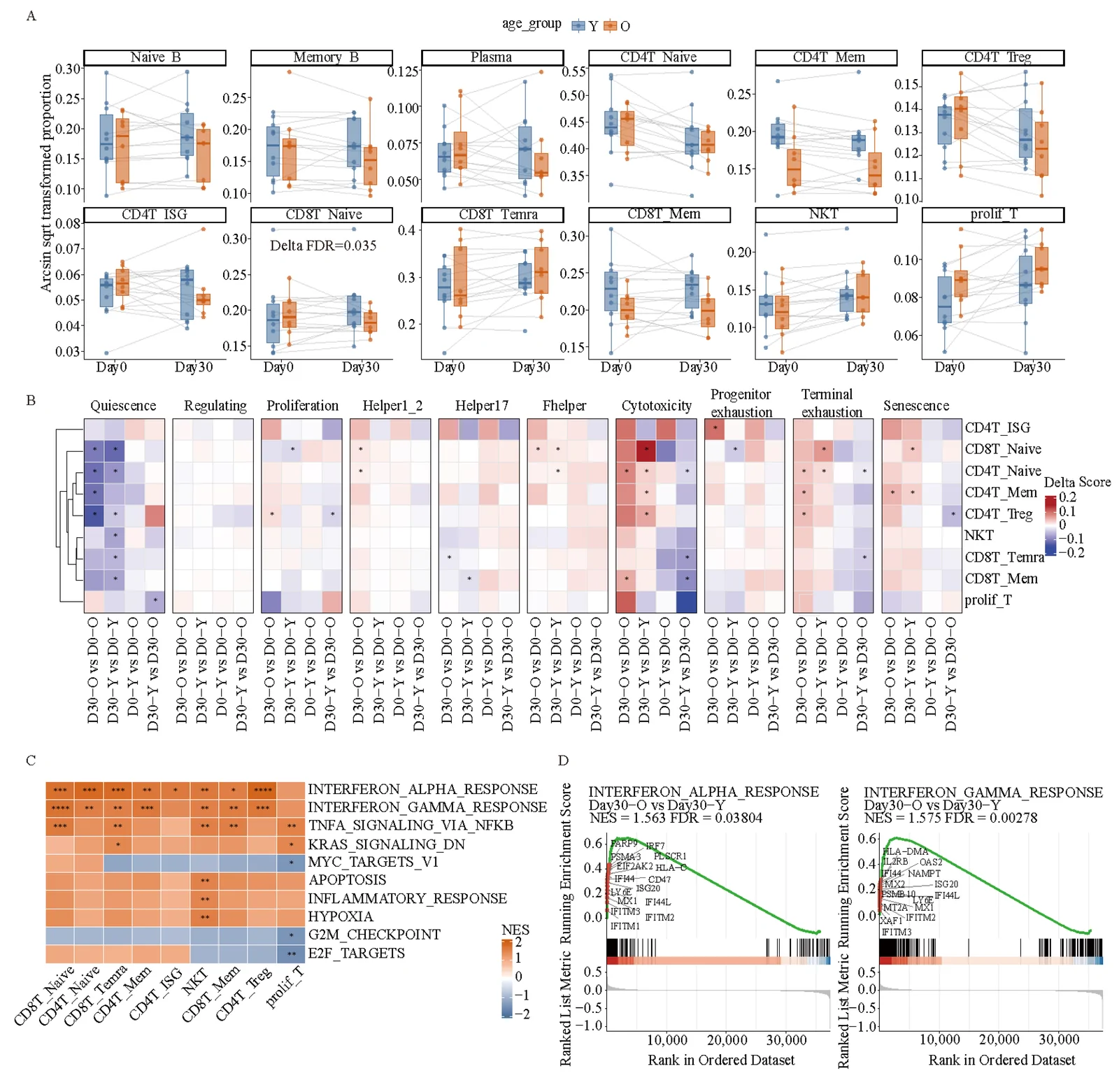
Differences in later adaptive immune states between age groups after VZV vaccination. (**A**) Abundance of 12 immune cell states at Day 0 and Day 30 in younger (Y; *n* = 10) and older (O; *n* = 8) participants. Proportions were arcsine square root transformed, and gray lines connect paired observations. Differences between age groups at each visit and in Day 30 minus Day 0 changes were assessed using Wilcoxon rank-sum tests with BH correction. (**B**) Differences in TCellSI functional scores across visits within each group and between the two groups. Tile color shows the score difference for each comparison. * indicates FDR < 0.05. (**C**) Hallmark gene set enrichment at Day 30 for older versus younger participants across T cell states. Tile color represents the normalized enrichment score. * FDR < 0.05, ** FDR < 0.01, *** FDR < 0.001, and **** FDR < 0.0001. (**D**) Enrichment plots for the interferon alpha and interferon gamma response gene sets in memory CD8 T cells at Day 30. TCellSI is the R package used to assess T cell states from gene expression data. Temra, terminally differentiated effector memory T cells; NES, normalized enrichment score; FDR, false discovery rate; BH, Benjamini–Hochberg; Y, younger participants aged <70 years; O, older participants aged ≥70 years.

We next examined differences between age groups in the later immune response at Day 30, focusing on the 12 cell states not included in the early innate immune analysis. No cell state differed between the groups at Day 0 or Day 30 after BH correction. Among the changes from Day 0 to Day 30 calculated for each participant, only naive CD8 T cells differed between the two groups. Their transformed abundance increased in younger participants and decreased slightly in older participants (Figure 6A; Table S11). Thus, the late compositional difference associated with age was selective rather than shared across T and B cell states.

TCellSI scores revealed broader differences in functional state than were evident from cell abundance. Several T cell states in both groups showed lower quiescence and higher cytotoxicity or terminal exhaustion scores at Day 30. In the direct age comparison at Day 30, cytotoxicity scores were higher in older participants in memory and Temra CD8 T cells, while the terminal exhaustion score was higher in Temra CD8 T cells. Older participants also had a higher senescence score in regulatory CD4 T cells. In proliferating T cells, quiescence was higher and proliferation was directionally lower in the older group (Figure 6B; Table S12). These scores reflect transcriptional signatures associated with T cell exhaustion and senescence.

Gene set enrichment analysis showed that these differences at Day 30 were associated with transcriptional programs related to interferon signaling. Interferon alpha and gamma response gene sets were positively enriched in older participants across several T cell states, and TNF signaling through NF-κB was also higher in selected states. In contrast, MYC targets, E2F targets, and the G2/M checkpoint were enriched in proliferating T cells from younger participants (Figure 6C). Within memory CD8 T cells, the interferon alpha response (NES = 1.563, FDR = 0.03804) and interferon gamma response (NES = 1.575, FDR = 0.00278) were enriched in the older group (Figure 6D; Table S13). At the regulatory level, E2F1 activity in naive CD8 T cells was higher in older participants at baseline, while JUND and JUN activities were higher at Day 30 (Figure S5A,B; Table S14).

CellChat identified differences among conditions in communication directed toward regulatory CD4 T cells. Inferred CD86–CD28 communication from classical monocytes to regulatory CD4 T cells was present at both Day 0 and Day 30 in the older group but was detected only at Day 0 in the younger group (Figure S6); the broader CD86 network is shown in Figure S5C. B cell functional modules and ligand–receptor results showed fewer differences associated with age, involving selected interferon response and antigen processing scores in naive B cells (Figure S5D; Table S15) and interactions that included CD55–ADGRE5 (Figure S5E). Overall, the later age associations were concentrated in CD8 T cell state and regulatory programs and in selected communication features, rather than in broad shifts in adaptive immune cell abundance.

## 4. Discussion

In this longitudinal study, conventional immunogenicity measurements in 100 participants were linked to early Olink profiles in 50 participants and longitudinal single-cell profiles in 18 participants. The findings support our hypothesis that early and later immune responses differ and that differences between younger and older adults involve selected immune cell populations. Both groups showed increased gE and VZV antibody levels by Day 30 (Figure 1B,C). The frequency of gE-specific IL2^+^ CD4T cells did not increase significantly in either group, whereas the frequency of gE-specific IFNγ^+^ CD4T cells increased significantly in younger but not older participants (Figure 1D,E). The molecular analyses add information about when and in which cell populations immune changes occur.

Zostavax, developed by Merck in the United States, was the first licensed live attenuated vaccine for preventing herpes zoster. Sullivan et al. summarized increases in VZV antibody levels in both younger and older adults, with antibody responses peaking two to four weeks after vaccination [37]. The increases in gE and VZV antibodies at Day 30 in our study are consistent with this pattern of humoral boosting after live vaccination.

Studies of Zostavax also provide context for the more variable cellular responses observed here. Johnson et al. found that only a subset of Zostavax recipients met the criteria for a gE-specific T cell response [38]. Our findings similarly showed a more limited pattern of gE-stimulated cytokine responses than of antibody increases. These observations suggest that humoral and cellular responses to live zoster vaccination need not increase in parallel.

Host immune competence is also relevant to the safety of live zoster vaccination. Kennedy and Grose reviewed serious adverse events after Zostavax, including vaccine virus dissemination in recipients with hematologic disease or immunosuppressive treatment [39]. These cases highlight the importance of cellular immunity in controlling vaccine virus replication. For the Chinese vaccine, Cao et al. found no increased hospitalization risk during the first 42 days compared with a later control period [40]. Although this population study does not exclude rare events, it provides complementary evidence on safety in routine use. Our immune measurements do not directly address vaccine virus replication or the mechanisms of serious adverse events.

Peripheral blood represents only one compartment of VZV immunity. Wang et al. identified VZV reactive memory CD4 T cells capable of producing multiple cytokines in paired blood and bone marrow samples [41]. They estimated that bone marrow contained a larger pool of these cells than peripheral blood. These findings place circulating T cell measurements within a broader view of VZV immune memory distributed across anatomical compartments.

The molecular profiles complement conventional immune measurements by resolving early responses within defined circulating cell states. Among the visits sampled, Day 1 showed the broadest transcriptional changes, particularly in classical monocytes and naive CD4 T cells. Previous systems analyses of Zostavax identified relationships among early transcriptional and metabolic changes and subsequent immune responses [14]. Our results add cellular resolution to these observations by identifying distinct responses within circulating monocyte, dendritic cell, and T cell states. Olink profiling also identified a shared early protein response, with exploratory differences between age groups. These complementary measurements describe early vaccine responses through both cellular transcription and circulating immune mediators.

In the phase 3 trial of this vaccine, efficacy estimates were 62.7%, 64.4%, and 18.6% among adults aged 50–59, 60–69, and ≥70 years, respectively. However, the estimate for the oldest group was imprecise (95% CI, −80.4% to 64.0%) [8]. These findings motivate closer examination of the quality and coordination of vaccine responses in older adults. In our study, older participants showed higher antiviral, interferon, or inflammatory signals in selected cell populations and circulating proteins. Such signals may reflect differences in baseline immune state, response regulation, or the persistence of activation, rather than more effective protective immunity. In an antigen-specific study, Sturmlechner et al. found that the durable response associated with Shingrix involved TH17 programs with less acquisition of Treg features than after Zostavax [42]. This provides a relevant framework for considering the balance between effector and regulatory differentiation. In our data, later CD8 T cell programs and inferred CD86–CD28 communication toward regulatory CD4 T cells identify possible components of that balance. These findings raise the possibility that increased activation and regulation coexist, although the functional contribution of Treg cells remains to be established.

The nested design and paired sampling allowed immune changes to be examined at several levels, but the small single-cell cohort limits comparisons between age groups. The sampling schedule could not resolve the onset, peak, and decline of antigen-specific cellular responses. Targeted protein measurements were restricted to Day 0 and Day 1. Although gE-stimulated CD4 T cell cytokines were measured, responses across the broader VZV antigen repertoire and the antigen specificity of the sequenced cells were not characterized. TCellSI scores, regulon activity, and CellChat networks require functional validation. No clinical protection or subsequent herpes zoster outcomes were assessed, so the observed molecular features cannot be designated as correlates of protection. Larger cohorts with additional sampling and antigen-specific assays are needed to test whether these features predict the magnitude or durability of vaccine responses.

This study identifies temporal changes and differences between age groups within circulating immune populations. These findings highlight early innate responses, later T cell states, and immune regulation as aspects of vaccine responsiveness that warrant further investigation. Determining how these features contribute to durable protection will require linking molecular profiles to functional immune responses and clinical outcomes.

## 5. Conclusions

Live attenuated herpes zoster vaccination induced clear humoral responses in both age groups, a broad molecular response at Day 1, and a more selective immune state at Day 30. The nested cohort design connected conventional immune measurements in 100 participants with early circulating protein responses in 50 participants and longitudinal single-cell programs in 18 participants. Molecular differences involved selected baseline pDC and non-classical monocyte features, the early cDC2 response, nominal early protein patterns, and later CD8 T cell and regulatory programs. These findings provide a framework for studying how immune activation and regulation vary with age after herpes zoster vaccination, but the proposed mechanisms require confirmation with larger cohorts, antigen-specific functional assays, longer follow-up, and clinical outcomes.

## Data Availability

All single-cell RNA sequencing data were deposited into the National Microbiology Science Data Center (https://nmdc.cn/), and the dataset ID is NMDC10020893. All other data supporting this study are available within the article. For additional information or requests, please contact the corresponding authors.

## Supplementary Materials

The following supporting information can be downloaded at: https://www.mdpi.com/article/10.3390/vaccines14100884/s1, Figure S1: Gating strategy for intracellular cytokine analysis of gE-stimulated CD4 T cells; Figure S2: Abundance of annotated immune cell states across visits; Figure S3: Age effects on regulon activity across innate immune cell states; Figure S4: Cell communication inferred by CellChat at Day 0 and Day 1; Figure S5: Regulatory programs and communication features of T and B cell states at Day 0 and Day 30; Figure S6: Communication directed to regulatory CD4 T cells at Day 0 and Day 30; Table S1: Flow cytometry antibody panels; Table S2: Participant characteristics and phenotype data; Table S3: Cell abundance; Table S4: Pseudobulk differentially expressed genes (DEGs); Table S5: Gene Ontology (GO) Biological Process (BP) enrichment; Table S6: Longitudinal blood transcription module (BTM) statistics; Table S7: Innate cell abundance; Table S8: Innate blood transcription module (BTM) age statistics; Table S9: Innate regulon activity; Table S10: Olink protein data and statistics; Table S11: Later adaptive cell abundance; Table S12: TCellSI scores; Table S13: Hallmark gene set enrichment analysis; Table S14: T cell regulon activity; Table S15: B cell functional module scores.
