# Supplementary material for: Temporal and Cellular Organization of Immune Responses to Live Attenuated Herpes Zoster Vaccination
Source: Vaccines (Basel). 2026 Oct 8;14(10):884. doi: 10.3390/vaccines14100884 (PMC13709950; doi:10.3390/vaccines14100884)
Supplement: Supplementary file 1 [file vaccines-14-00884-s001.zip › Supplementary_Figures_S1-S6.pdf]

# Supplementary Materials

## Temporal and Cellular Organization of Immune Responses to Live Attenuated Herpes Zoster Vaccination

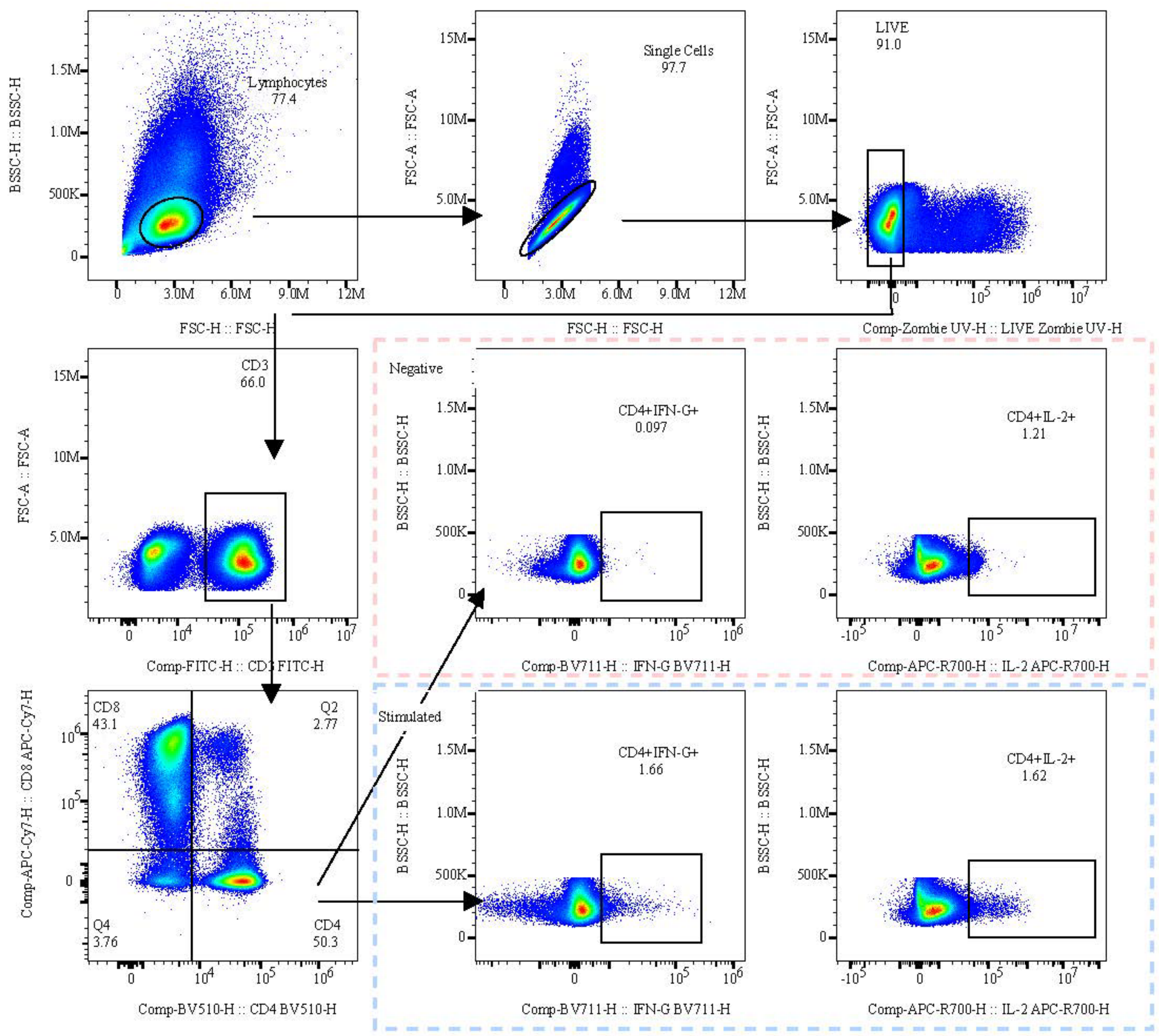


**Figure S1.** (A) Gating strategy for intracellular cytokine analysis of gE stimulated CD4 T cells.

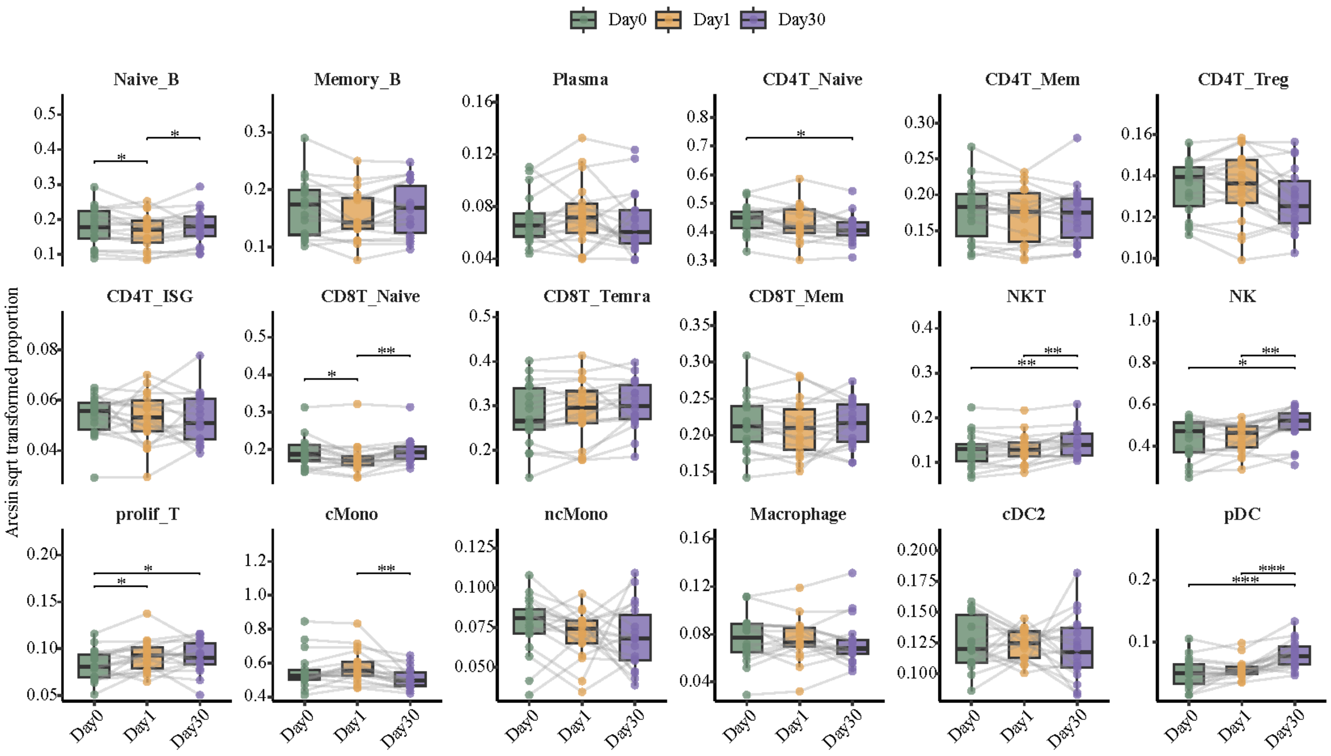


**Figure S2.** (A) Abundance of all 18 annotated immune cell states at Day 0, Day 1, and Day 30. Proportions were arcsine square root transformed. Points represent participants, gray lines connect paired observations, and boxes show the median and interquartile range. Differences between visits were assessed using paired Wilcoxon signed-rank tests with BH correction. *$FDR$ < 0.05, **$FDR$ < 0.01, and ***$FDR$ < 0.001.

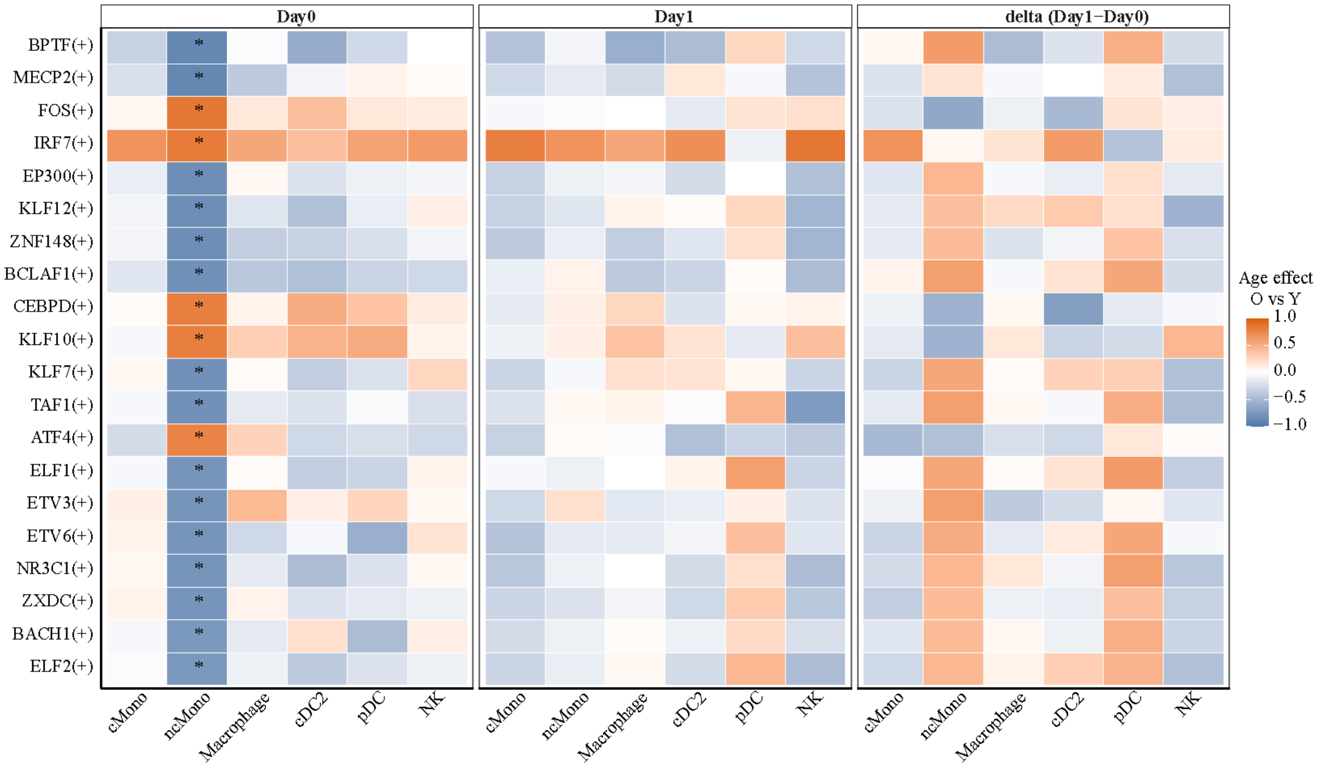


**Figure S3.** (A) Age effects on regulon activity across innate immune cell states at Day 0, Day 1, and for Day 1 minus Day 0 changes. Tile color represents Cliff's delta for O versus Y. Positive and negative values indicate higher regulon activity in the older and younger groups, respectively. Stars indicate *FDR* < 0.05 after BH correction.

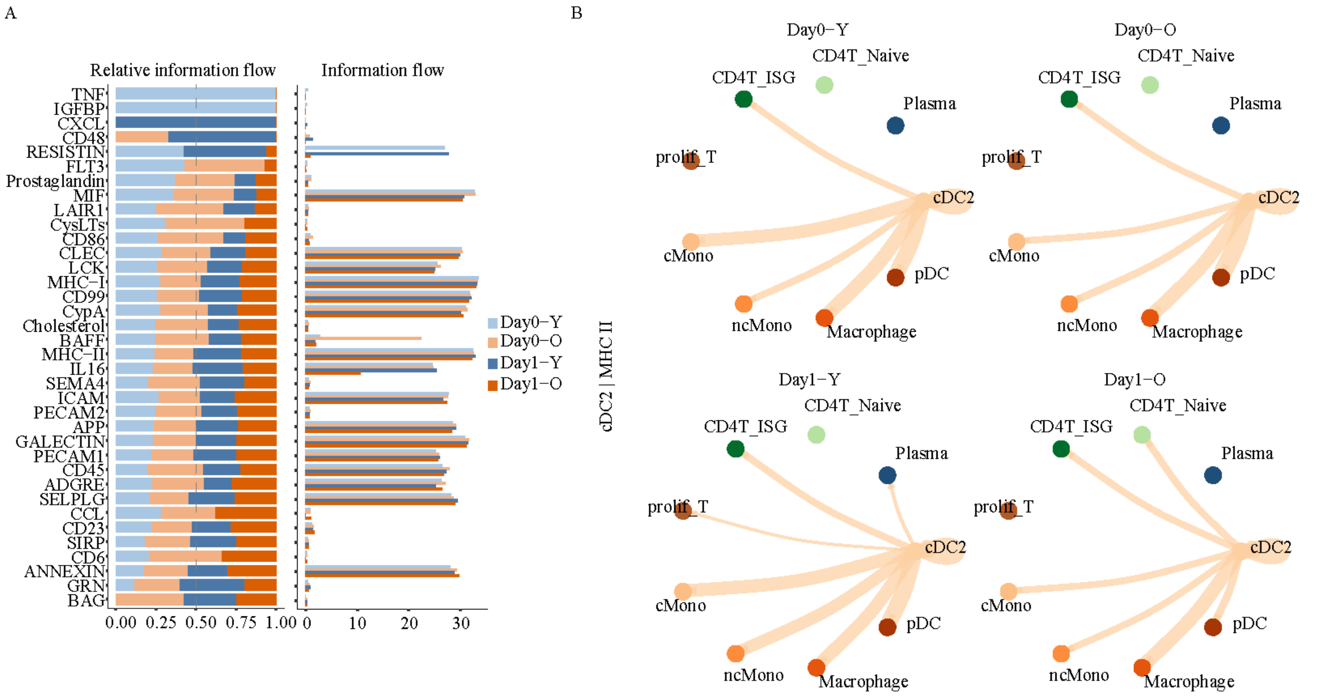


**Figure S4.** Cell communication inferred by CellChat at Day 0 and Day 1. (A) Relative and absolute information flow across signaling pathways in younger and older participants. (B) MHC-II signaling networks originating from cDC2. Arrows indicate signaling direction, and line width represents inferred communication strength. Y, younger participants aged <70 years; O, older participants aged ≥70 years; cDC2, conventional dendritic cell type 2.

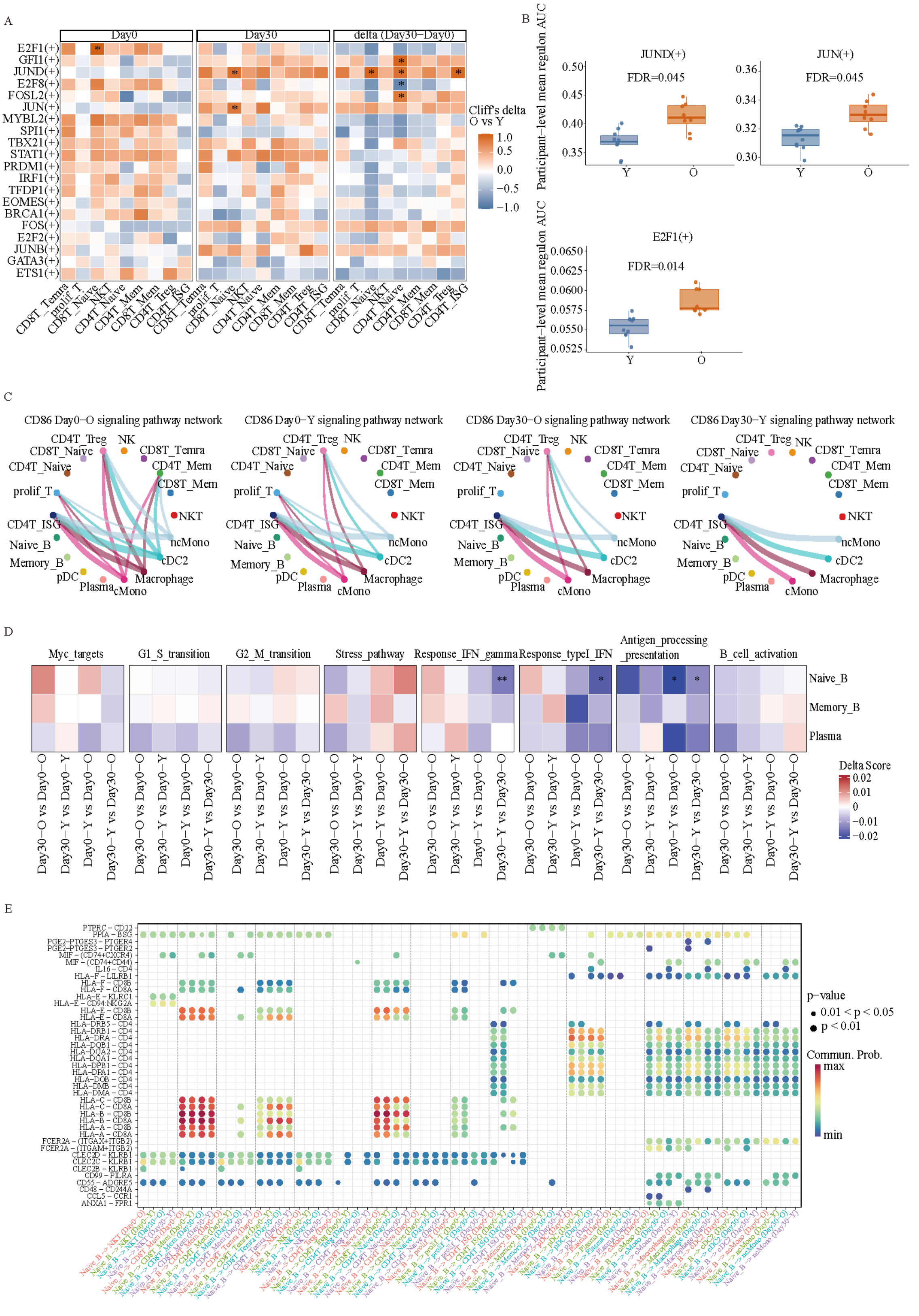


**Figure S5.** Regulatory programs and communication features of T and B cell states at Day 0 and Day 30. (A) Cliff's delta for T cell regulon activity at Day 0, Day 30, and for Day 30 minus Day 0 changes. Positive and negative values indicate higher activity in the older and younger groups, respectively. * indicates *FDR* < 0.05 after BH correction. (B) AUC values for E2F1, JUND, and JUN regulons in naive CD8 T cells summarized for each participant. Differences between age groups were assessed using Wilcoxon rank-sum tests with BH correction. (C) CellChat inferred CD86 signaling networks in the younger and older groups at Day 0 and Day 30. Arrows indicate signaling direction, and line width represents inferred communication strength. (D) Differences in functional module scores across naive B cells, memory B cells, and plasma cells for within-group visit comparisons and between-group comparisons. Tile color shows the score difference for the indicated comparison. *$FDR$ < 0.05 and **$FDR$ < 0.01 after BH correction. (E) Ligand–receptor communication inferred by CellChat and originating from naive B cells. Dot color represents communication probability, and dot size represents the *P* value category.

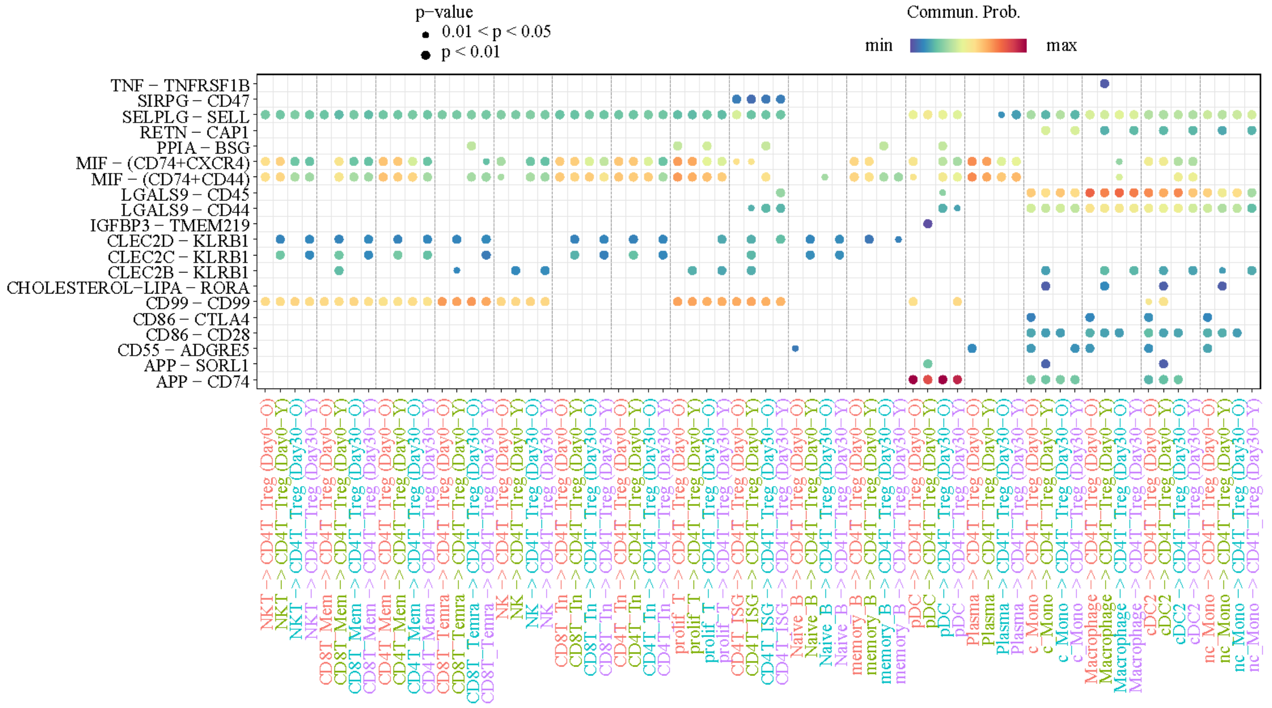


**Figure S6.**(A) Communication directed to regulatory CD4 T cells at Day 0 and Day 30. Ligand‑receptor communication was inferred using CellChat in younger and older participants. Dot color represents communication probability, and dot size represents the P value category. Y, younger participants aged <70 years; O, older participants aged ≥70 years.
